# A new perspective on the ^137^Cs retention mechanism in surface soils during the early stage after the Fukushima nuclear accident

**DOI:** 10.1038/s41598-019-43499-7

**Published:** 2019-05-07

**Authors:** Jun Koarashi, Syusaku Nishimura, Mariko Atarashi-Andoh, Kotomi Muto, Takeshi Matsunaga

**Affiliations:** 1 Nuclear Science and Engineering Center, Japan Atomic Energy Agency, Ibaraki, 319-1195 Japan; 2 Tono Geoscience Center, Japan Atomic Energy Agency, Gifu, 509-5102 Japan

**Keywords:** Environmental chemistry, Environmental impact

## Abstract

The Fukushima Daiichi nuclear power plant accident caused serious radiocesium (^137^Cs) contamination of the soil in multiple terrestrial ecosystems. Soil is a complex system where minerals, organic matter, and microorganisms interact with each other; therefore, an improved understanding of the interactions of ^137^Cs with these soil constituents is key to accurately assessing the environmental consequences of the accident. Soil samples were collected from field, orchard, and forest sites in July 2011, separated into three soil fractions with different mineral–organic interaction characteristics using a density fractionation method, and then analyzed for ^137^Cs content, mineral composition, and organic matter content. The results show that 20–71% of the ^137^Cs was retained in association with relatively mineral-free, particulate organic matter (POM)-dominant fractions in the orchard and forest surface soil layers. Given the physicochemical and mineralogical properties and the ^137^Cs extractability of the soils, ^137^Cs incorporation into the complex structure of POM is likely the main mechanism for ^137^Cs retention in the surface soil layers. Therefore, our results suggest that a significant fraction of ^137^Cs is not immediately immobilized by clay minerals and remains potentially mobile and bioavailable in surface layers of organic-rich soils.

## Introduction

The accident at the Fukushima Daiichi nuclear power plant (NPP) in March 2011 released substantial amounts of radionuclides into the atmosphere and consequently caused serious radioactive contamination of terrestrial ecosystems over a wide area of eastern Japan^1^. Of the radionuclides found in these terrestrial ecosystems, ^137^Cs, with a physical half-life of 30.1 years, is the primary source of concern because of its potential radiological impact on humans and the ecosystems over the coming decades.

The behavior of ^137^Cs in soil after deposition is one of the key factors necessary to evaluate the long-term radiation risks that may be caused by external and internal exposure. It is generally accepted that, once ^137^Cs reaches the surface layers of the mineral soil, it is strongly adsorbed (i.e., fixed) by clay minerals^2,3^, rapidly reducing the mobility and bioavailability of ^137^Cs in such soils^4–10^. Konopleva *et al*.^11^ demonstrated that, 19 years after the Chernobyl NPP accident, more than 50% of the Chernobyl-derived ^137^Cs remained in the upper 10-cm soil layer in German forest ecosystems. The long-term accumulation of Chernobyl-derived ^137^Cs in the surface soil layers has also been observed in grassland ecosystems in European countries^12,13^. In Japan, it has recently been reported that, at a Japanese cedar plantation even 38 years after the fallout from atmospheric nuclear weapons testing, the deposited ^137^Cs was still observed, primarily in the uppermost 5 cm of the mineral soil^14^. In addition, there are many observations showing that ^137^Cs in mineral soil is more highly concentrated in clay-sized (<2 μm) soil particles than in larger-sized soil particles, reflecting the specific adsorption of ^137^Cs onto clay minerals^15–19^. These studies support the overall immobility of ^137^Cs in surface soil layers over a long period of time after deposition as a result of the interaction of ^137^Cs with clay minerals in the soil layers.

However, there are also studies suggesting that the ^137^Cs behavior in the mineral soil cannot simply be evaluated based on the interaction of ^137^Cs with clay minerals and there are many factors that can affect the mobility and bioavailability of ^137^Cs in soils. These factors, other than the clay mineralogy, include soil physicochemical properties such as the pH, cation-exchange capacity (CEC), presence of exchangeable cations, organic matter content^9,20–23^, and soil structure^24–26^, soil microbial activities^27–29^, and local climatic and ecological conditions^30–32^.

Soil is a complex system where soil minerals, organic matter, and microorganisms interact with each other in various ways. These soil constituents vary greatly in their quantities, qualities, and properties depending on the site. Therefore, it is highly likely that the interactions of ^137^Cs with these soil constituents, and therefore the predominant mechanisms of ^137^Cs retention induced by these interactions in the surface soil layers, also vary between sites. This underlines the importance of investigations under the conditions of the specific ecosystems in the areas of interest, that is, investigations in the actual areas affected by the Fukushima NPP accident are required to accurately assess the environmental consequences of the Fukushima NPP accident.

Soil organic matter has both direct and indirect influences on the ^137^Cs behavior in mineral soils. Soil organic matter provides a high CEC and is therefore capable of adsorbing ^137^Cs; however, this adsorption is not specific to ^137^Cs and adsorbed ^137^Cs is easily exchangeable with other cations^20,22^. In addition to direct influences, an indirect influence of soil organic matter on the ^137^Cs behavior has also been suggested in that the presence of soil organic matter inhibits the fixation of ^137^Cs by clay minerals by limiting the access of ^137^Cs to ^137^Cs-fixation sites on the clay minerals^22,33^. It has recently been shown that soil aggregation (i.e., the binding of soil particles into aggregates together with soil organic matter) has a significant influence on the mobility and bioavailability of ^137^Cs in mineral soils in Japanese forest ecosystems affected by the Fukushima NPP accident^26^. Given that soils (forest soils in particular) in Japan are rich in organic matter^34^ and soil organic matter is a dynamic constituent that turns over constantly via litter input and microbial decomposition, an improved understanding of the interactions between ^137^Cs, minerals, and organic matter is likely critical to predict the fate of Fukushima-derived ^137^Cs in terrestrial ecosystems in Japan.

**Figure 1:**
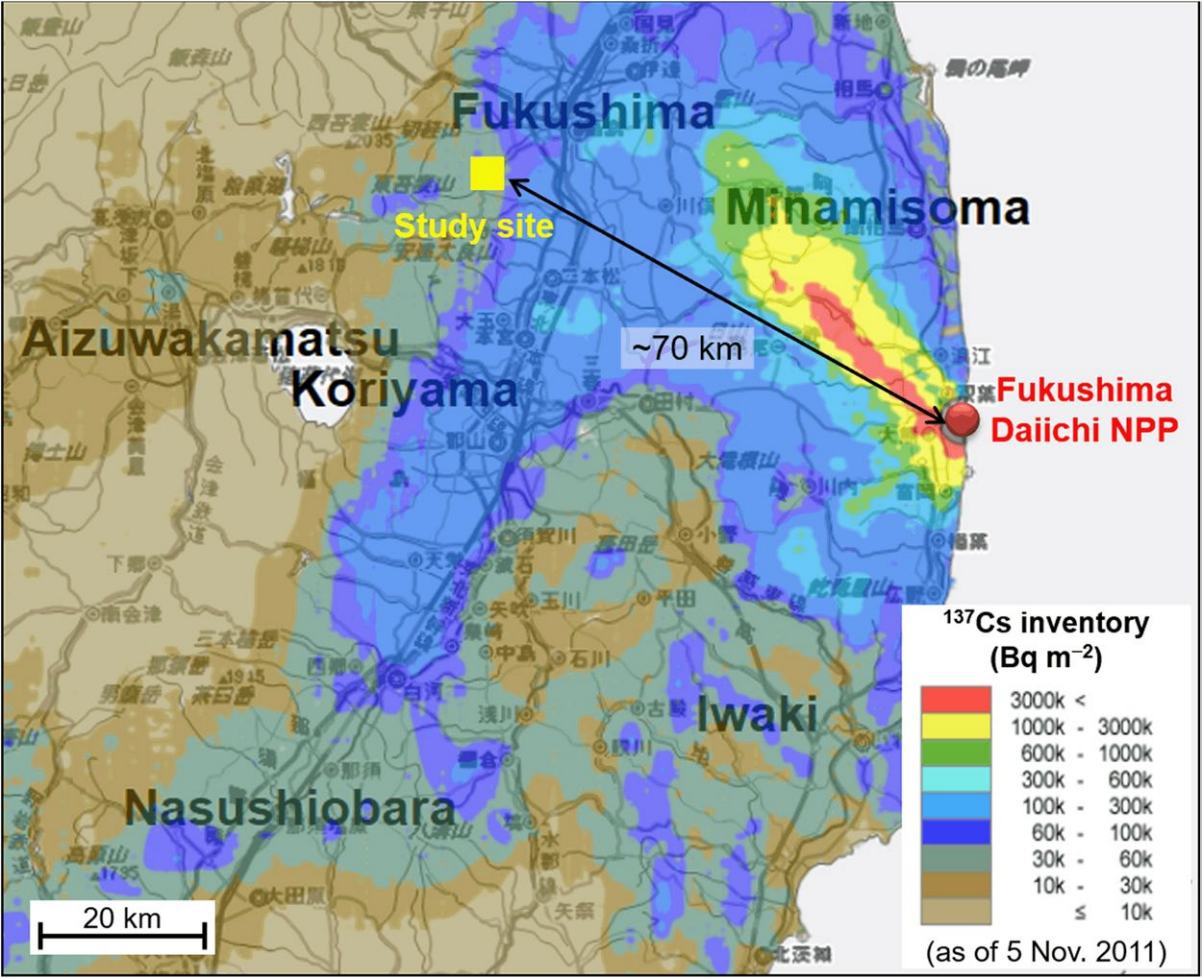
Location of the study site. The ^137^Cs inventory map was generated using the website “Extension Site of Distribution Map of Radiation Dose, etc.,/GIS Maps” prepared by the Ministry of Education, Culture, Sports, Science, and Technology, Japan^1^.

The aim of the present study is therefore to explore the retention mechanisms of ^137^Cs in the surface soil layers of terrestrial ecosystems affected by the Fukushima NPP accident, with a specific focus on the interactions between ^137^Cs, soil minerals, and organic matter. We collected surface soil samples from three types of terrestrial ecosystems (a field, CP-2; an orchard, CP-6; and a forest, FR-4) in July 2011 (approximately 3.5 months after the accident) and at the FR-4 site in July 2015 in the city of Fukushima (Fig. 1). The soil samples were separated into three soil fractions with different physical characteristics, a free particulate organic matter (POM)-dominant low-density fraction (fLF); a mineral-associated low-density fraction (mLF); and a mineral-dominant high-density fraction (HF), using a density fractionation method^35–37^. The soil fractions were then analyzed for their ^137^Cs content, mineral composition, and organic matter (carbon and nitrogen) content.

## Results

### General properties of the soil samples

Selected physicochemical properties and ^137^Cs activity concentrations of the soil samples used in the present study are given in Table 1. The soils at the three sites had similar particle-size distributions. The forest soils (FR-4) were generally higher in organic C content and CEC than the cropland soils. The fractions of ^137^Cs extracted with 1 M ammonium acetate (NH_4_Ac, pH 7) from the CP-2 and FR-4 soil samples were approximately 10–19%^9^. Radiocesium in this fraction has been considered to be easily exchangeable with other cations such as NH_4_^+^ present in soil solution.

**Table 1:** Physicochemical properties and ^137^Cs activity concentrations of the soils investigated in the present study.

Date	Site	Depth (cm)	Soil texture (%)^a^			pH^a^	CEC^a^ (cmol kg^−1^)	Organic C (gC kg^−1^)	^137^Cs conc.^b^ (Bq kg^−1^ dw)	^137^Cs extractability^c^ (%)
			Sand	Silt	Clay					
July 2011	CP-2	0–1	43.7	38.6	17.7	6.2	18.4	16	1400 (200)^d^	12.1
		1–3	41.6	40.4	18.0	6.2	22.1	16	1400 (400)	9.9
	CP-6	0–1	48.1	36.2	15.7	5.6	28.9	96	6700 (600)	NA^e^
		1–3	57.4	26.8	15.8	5.3	29.1	65	1070 (70)	NA
		3–5	57.0	27.4	15.6	5.3	27.3	50	180 (30)	NA
	FR-4	0–1	49.9	34.7	15.4	5.6	42.6	194	2000 (300)	11.4
		1–3	51.5	31.8	16.7	5.5	35.6	136	470 (90)	19.0
		3–5	51.1	33.4	15.5	5.4	32.6	97	150 (30)	15.7
July 2015	FR-4	0–1	NA^e^	NA	NA	NA	NA	186	5690 ± 80^f^	NA
		2–3	NA	NA	NA	NA	NA	119	800 ± 10	NA
		4–5	NA	NA	NA	NA	NA	83	149 ± 2	NA

^a^Data are from Koarashi *et al*.^31^.

^b^Activity concentrations of ^137^Cs were corrected for radioactive decay to the sampling date.

^c^Fractions of ^137^Cs extracted with 1 M ammonium acetate (NH_4_Ac, pH 7) from the soil samples. Data are from Matsunaga *et al*.^9^.

^d^Mean and standard deviation (in parentheses) of the three replicate samples (N = 3).

^e^NA: Not available.

^f^Errors represent the counting errors in the radiation measurement.

### Microscopic observations of the soil physical fractions

**Figure 2:**
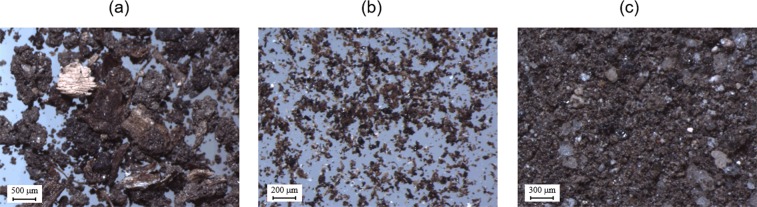
Examples of (**a**) fLF, (**b**) mLF, and (**c**) HF fraction samples isolated from the topmost (0–1 cm) mineral soil layer at the forest (FR-4) site observed using a stereomicroscope.

The physical fractionation at a density of 1.6 g cm^−3^ successfully separated the soil samples into three fractions (fLF, mLF, and HF) showing different physical characteristics (Fig. 2). The fLF fraction consisted primarily of plant detritus or POM with recognizable structures. It also appeared that some of the POM in the fLF fraction was coated with fine mineral particles and organic materials. The mLF fraction consisted of finely fragmented organic materials (typically less than 100 μm in size) with less recognizable structures. Some larger-sized plant residues were also observed in the mLF fraction. The HF fraction was dominated by soil mineral particles, some of which appeared to be associated with very fine organic materials.

### Mass distributions of the soil physical fractions

**Figure 3:**
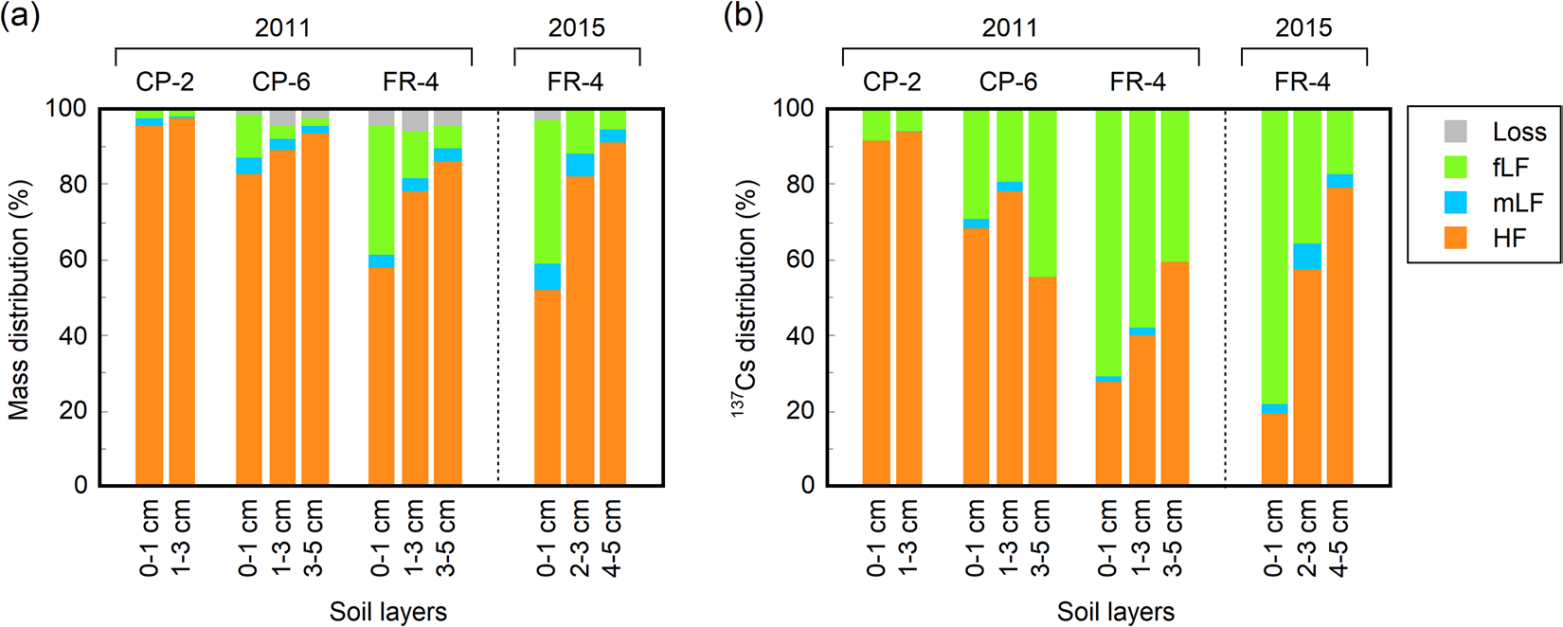
Distributions of the (**a**) soil mass and (**b**) ^137^Cs inventory in the three physical fractions of the surface mineral soil layers at the field (CP-2), orchard (CP-6), and forest (FR-4) sites.

In general, the HF fraction accounted for the largest proportion (52–97%) of the soil mass at all sites and depths; however, the mass distribution patterns of the three physical fractions differed between the sites for the different land-use conditions (Fig. 3a). At the field (CP-2) site, the HF fraction made up more than 95% of the soil mass and was independent of the soil depth. At the forest (FR-4) site, the fLF fraction represented a significant proportion (34–38%) of the soil mass in the topmost (0–1 cm) layers and the mass proportion of the fLF fraction decreased with depth to 5–6% in the lowest soil layers. The orchard (CP-6) site also showed a depth-wise trend for the mass distribution similar to that at the FR-4 site; however, the mass proportion of the fLF fraction was smaller than that at the FR-4 site throughout the soil profile. At all sites, the mLF fraction comprised a minor fraction (0.6% up to 7.1%) of the soil mass and showed no consistent decrease or increase in the mass proportion with depth.

### ^137^Cs activity concentrations of the soil physical fractions

For all of the soil samples collected in 2011, the fLF fractions showed the highest ^137^C activity concentration of the three physical fractions (Table 2). The ^137^Cs activity concentrations of the mLF and HF fractions were similar and were 2–10 times lower than those of the fLF fraction. This distribution pattern of the ^137^Cs activity concentrations also held for the soil samples collected in 2015 at the forest (FR-4) site.

**Table 2:** ^137^Cs activity concentrations of the three physical fractions of the soils.

Date	Site	Depth (cm)	^137^Cs concentration (Bq kg^−1^ dw)^a^		
			fLF	mLF	HF
July 2011	CP-2	0–1	3290 ± 110^b^	<600^c^	1010 ± 20^b^
		1–3	2830 ± 80	490 ± 140^b^	940 ± 30
	CP-6	0–1	17500 ± 200	3970 ± 50	5560 ± 110
		1–3	3870 ± 120	510 ± 30	600 ± 20
		3–5	4780 ± 150	<90^c^	148 ± 5
	FR-4	0–1	3480 ± 70	880 ± 40	810 ± 30
		1–3	1560 ± 50	180 ± 20	170 ± 5
		3–5	920 ± 40	<60^c^	94 ± 5
July 2015	FR-4	0–1	10550 ± 40	1620 ± 40	1928 ± 7
		2–3	2080 ± 20	850 ± 30	484 ± 7
		4–5	300 ± 20	100 ± 40	83 ± 5

^a^Activity concentrations of ^137^Cs were corrected for radioactive decay to the sampling date.

^b^Errors represent the counting errors in the radiation measurement.

^c^The ^137^Cs concentration was below the lowest detectable concentration.

### ^137^Cs distribution between the soil physical fractions

Due to the unequal ^137^Cs concentrations of the three physical fractions (Table 2), the ^137^Cs distribution between the physical fractions showed a pattern that was very different from that of the mass distribution between the fractions (Fig. 3b). At the FR-4 site, more than 70% of the ^137^Cs was associated with the fLF fraction in the topmost soil layers in both years and the contribution of the fLF to the ^137^Cs retention was also remarkable (17–58%) in the underlying soil layers. Similarly, at the CP-6 site, the fLF fraction retained a significant proportion (20–45%) of the ^137^Cs in the soil. At the CP-2 site, however, fLF-associated ^137^Cs comprised 9% or less of the soil ^137^Cs and most of the ^137^Cs was contained within the HF fraction. The mLF fraction was a minor (<7%) fraction with respect to the retention of ^137^Cs in the soil across all sites.

### POM versus mineral particles in the fLF fractions

Via the ^137^Cs analysis of the soil physical fractions, it was found that the fLF fractions had the highest ^137^Cs activity concentration and consequently retained a significant proportion of the ^137^Cs in the soil. In addition, microscopic observations showed that the fLF fractions were composed primarily of POM including plant detritus; however, some of the POM appeared to be coated with fine mineral particles. To explore the mechanisms for the high ^137^Cs retention in the fLF fractions, we further separated the fLF fractions into two additional fractions, POM (fLF-POM) and mineral particles (fLF-MP), using a fractionation at a density of 1.9 g cm^−3^ after ultrasonic washing.

**Figure 4:**
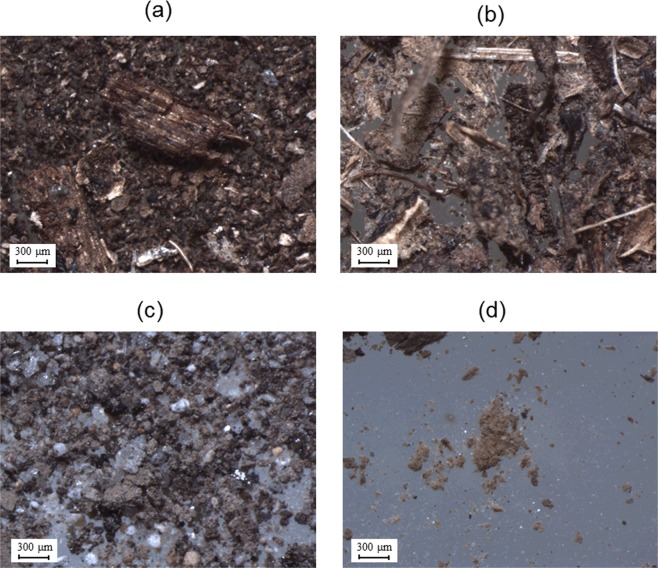
Examples of the fLF-POM and fLF-MP fraction samples isolated from the topmost (0–1 cm) mineral soil layers observed using a stereomicroscope: (**a**) fLF-POM at the forest (FR-4) site; (**b**) fLF-POM at the orchard (CP-6) site; (**c**) fLF-MP at the FR-4 site; and (**d**) fLF-MP at the field (CP-2) site.

Even though POM appeared to be the primary component of the fLF fractions in the topmost soil layers (Table 3, Fig. 4), there was also a non-negligible amount (14–28% on a mass fraction basis) of mineral particles (with a density greater than 1.9 g cm^−3^) in the fractions. At the CP-2 site, the ^137^Cs activity concentration was much higher in fLF-MP than in fLF-POM. Conversely, at the FR-4 site, the ^137^Cs activity concentration was an order of magnitude greater in fLF-POM than in fLF-MP. At the CP-6 site, the ^137^Cs activity concentration was similar in the two fractions. As a result, more than 90% of the fLF-associated ^137^Cs was found in fLF-MP for the CP-2 site, whereas for the CP-6 and FR-4 sites, more than 80% of the fLF-associated ^137^Cs was found in fLF-POM.

**Table 3:** ^137^Cs distribution between the fLF-POM and fLF-MP fractions.

Site	Depth (cm)	Mass distribution (%)			^137^Cs concentration (Bq kg^−1^ dw)		^137^Cs distribution (%)^a^	
		fLF-POM	fLF-MP	Loss	fLF-POM	fLF-MP	fLF-POM	fLF-MP
CP-2	0–1	55.7	28.0	16.3	730 ± 90^b^	13900 ± 600^b^	9.4	90.6
CP-6	0–1	78.7	14.3	7.0	14200 ± 400	17800 ± 700	81.4	18.6
FR-4	0–1	59.6	23.4	17.0	4200 ± 100	540 ± 130	95.2	4.8

^a^The fraction lost during the separation procedure was not considered when determining the ^137^Cs distribution in the fLF fraction.

^b^Errors represent the counting errors in the radiation measurement.

Across all sites, the fLF-POM fractions in the topmost soil layers were characterized by a high C content and C/N ratio (Table 4), indicating that the fractions were rich in relatively fresh, less degraded organic materials^38,39^. Conversely, the fLF-MP fractions generally had low C contents and C/N ratios, indicating that these fractions were dominated by soil mineral materials with a relatively small contribution of more degraded organic materials. These results were consistent with the visual inspection of fLF-POM and fLF-MP (Fig. 4).

**Table 4:** Carbon and nitrogen content of the fLF-POM and fLF-MP fractions.

Site	Depth (cm)	fLF-POM			fLF-MP		
		C (%)	N (%)	C/N	C (%)	N (%)	C/N
CP-2	0–1	36.0	2.1	17.2	7.0	0.8	9.3
CP-6	0–1	35.9	2.5	14.4	3.6	0.4	9.1
FR-4	0–1	31.9	1.8	18.0	1.0	0.1	11.6

**Figure 5:**
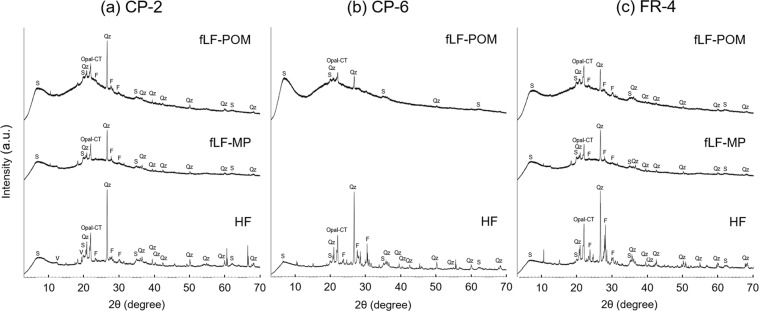
X-ray diffraction (XRD) patterns for the fLF-POM, fLF-MP, and HF fractions isolated from the topmost (0–1 cm) mineral soil layers at the field (CP-2), orchard (CP-6), and forest (FR-4) sites. Abbreviations: S, smectite; V, vermiculite; Qz, quartz; and F, feldspar.

The X-ray diffraction (XRD) patterns of the fLF-POM and fLF-MP fractions are given in Fig. 5 and compared to those of the corresponding HF fractions. The major mineral compositions in the soil fractions were quartz, feldspar, and smectite, and these minerals were observed even for the fLF-POM fractions. In general, the mineral compositions were very similar for the three fractions within each site. Minerals specific to radiocesium adsorption (e.g., micaceous clay minerals) were not identified in the fLF-POM fractions at any of the sites.

## Discussion

There has long been a general consensus that once ^137^Cs is transferred to the mineral soil, it is rapidly and nearly irreversibly fixed by clay minerals^2,40,41^. The fixation of ^137^Cs by clay minerals results in limited migration through the soil profile due to chemical and biological processes^10,12–14^ and, consequently, a reduced availability for ^137^Cs uptake by plants^11,42,43^. According to this consensus, Fukushima-derived ^137^Cs in the upper layers of mineral soil is assumed to be largely associated with soil fractions dominated by mineral particles; however, this was not the case in the present study. Most of the ^137^Cs was indeed associated with the mineral-dominant HF fraction at the CP-2 site; however, at the CP-6 and FR-4 sites where the fLF fraction had abundantly accumulated, the ^137^Cs was largely associated with the fLF fraction and this was also observed more than 4 years after the accident (Fig. 3).

Clearly, the most interesting question here is how such a large amount of ^137^Cs can be retained in the fLF fraction in the upper layers of the mineral soil at the fLF-rich cropland (CP-6) and forest (FR-4) sites. Four possible mechanisms merit consideration: (1) nonspecific adsorption by organic materials; (2) incorporation into the POM structure; (3) association with admixed clay minerals; and (4) retention in the soil microbial biomass.

According to mechanism (1), the ^137^Cs is largely adsorbed onto nonspecific cation-exchange sites provided by organic materials in the fLF fraction^20,22,44^. The soils at the CP-6 and FR-4 sites are rich in organic materials (organic C) and therefore have a high CEC compared to the CP-2 site (Table 1). However, the fractions of ^137^Cs associated with fLF in the soil samples (40–71% of the total ^137^Cs for the FR-4 site, Fig. 3b) were much larger than the fractions of ^137^Cs extracted with 1 M ammonium acetate (NH_4_Ac, pH 7) from the samples (11–19%, Table 1). This indicates that most of the fLF-associated ^137^Cs was present in a form that is not easily exchangeable with other cations, which leads us to reject nonspecific adsorption as the main mechanism for the ^137^Cs retention in the fLF fraction.

According to mechanism (2), the ^137^Cs is physically trapped in complex structures of degraded POM (Fig. 4) or incorporated into the tissues of fragmented plant debris (dead cedar leaves) in the fLF fraction, which makes the ^137^Cs unextractable via the ion-exchange reaction. This mechanism is partly supported by the present result showing that the fLF-associated ^137^Cs primarily (81–95%) resides in the fLF-POM fraction that was obtained after ultrasonically washing the fLF fraction to remove the attached mineral particles for the CP-6 and FR-4 sites (Table 3). This mechanism is also consistent with the finding of Tanaka *et al*.^45^, who showed that ^137^Cs was incorporated into and strongly fixed in the interior tissues of dead cedar leaves that fell on the forest floor prior to the Fukushima NPP accident. Kato *et al*.^46^ showed that, in Japanese cedar forests, the deposition of ^137^Cs from the tree canopy to the forest floor occurred primarily via throughfall, not litterfall (i.e., as ^137^Cs incorporated into the leaf tissues), during the early stage (<200 days) after the Fukushima NPP accident. Therefore, the incorporation of ^137^Cs into the POM structure after deposition to the mineral soil as a dissolved form appears to be a plausible mechanism for ^137^Cs retention in the fLF fraction as of July 2011.

According to mechanism (3), the ^137^Cs is actually fixed in the clay minerals and the clay minerals are tightly associated with POM^26,47^ or rather incorporated into the POM structure. Indeed, the XDR analysis showed that minerals were still present in fLF-POM (Fig. 5). However, the mineral composition of fLF-POM was similar to that of fLF-MP, as well as that of HF, and minerals specific to ^137^Cs adsorption (e.g., micaceous minerals) were not found in the fLF-POM fraction. In addition, in the FR-4 soil, the ^137^Cs activity concentration of fLF-POM was much higher than those of fLF-MP and HF (Tables 2 and 3). Under these circumstances, this mechanism can only be acceptable if a selective association occurs between POM and ^137^Cs-concentrated clay minerals in the soil. However, this is unlikely.

According to mechanism (4), the ^137^Cs is retained in the soil microbial biomass in the fLF fraction. Microorganisms in soils have been found to be able to accumulate ^137^Cs^48–50^. Given that soil microorganisms are generally abundant in organic-rich surface soil layers and that fLF fractions are deficient in clay minerals, which are a strong adsorbent of ^137^Cs, it is conceivable that soil microorganisms are responsible for the retention of ^137^Cs, particularly in the fLF fraction. Brückmann and Wolters^27^ have shown that, on average, 13% (with a large variability between 1% and 56%) of the ^137^Cs inventory was immobilized in the microbial biomass in forest-floor organic layers of German forest ecosystems. However, Stemmer *et al*.^28^ reported that the amount of microbial biomass-bound ^137^Cs was less than 2% of the ^137^Cs inventory in the surface 0–3 cm of mineral soil in an alpine meadow in Austria. Similarly, a minor contribution of the microbial biomass to ^137^Cs retention has been reported for subtropical forest soils (0–10-cm depth) in Taiwan^29^. Following the Fukushima NPP accident, it has been shown that the uptake of ^137^Cs by soil microorganisms is less important for the retention of ^137^Cs in forest surface (0–3 cm) soils in Fukushima compared to ion-exchange adsorption on nonspecific sites provided by abiotic components^8^. In light of these studies, this mechanism may be partly responsible for the ^137^Cs retention in the fLF fraction; however, it is clearly not entirely sufficient to explain the observed amount of fLF-associated ^137^Cs (20–78% of the total ^137^Cs, Fig. 3b) in the soils at the CP-6 and FR-4 sites.

The fLF fraction is relatively less associated with and protected by soil minerals; accordingly, this fraction is considered more easily degradable by soil microorganisms than other fractions^51–53^. Therefore, this fraction possibly offers a large but temporary reservoir of ^137^Cs in soils. As the microbial decomposition of fLF proceeds, the ^137^Cs retained in this fraction can be mobilized, become available to plants, and have the opportunity to be fixed by clay minerals (i.e., to move into the HF fraction) or to migrate downward through the soil profile. In the present study, however, the proportion of fLF-associated ^137^Cs to the total ^137^Cs inventory did not decrease between July 2011 (3.5 months after the accident) and July 2015 (4 years later) at the forest site (Fig. 3b). A similar observation has been reported for surface mineral soils at two forest sites in Fukushima between September 2012 and September 2014^44^. This may be due to the continuous supply of ^137^Cs-contaminated POM from the forest-floor organic layer to the mineral soil during the first four years following the accident. Supporting this, studies conducted since the Fukushima NPP accident have shown a rapid (within several years) migration of ^137^Cs from organic layers to the mineral soil in Japanese forest ecosystems^10,54–59^. Therefore, it is hypothesized that, at the CP-6 and FR-4 sites, ^137^Cs mobilization from the fLF fraction via microbial degradation was compensated for by the ^137^Cs supply (as POM) from the organic layers during this period. If this is true, the fLF-associated ^137^Cs will decrease with time and with decreasing ^137^Cs inventory in the organic layers and the HF-associated ^137^Cs will become important in controlling the mobility and bioavailability of ^137^Cs in the surface mineral soils.

The mLF fraction had a low ^137^Cs activity concentration and a low ^137^Cs inventory compared to the other two fractions (Table 2 and Fig. 3), indicating that finely fragmented organic materials strongly associated with soil minerals (although some might have been occluded in soil aggregates) have a low ^137^Cs retention capability. This is in stark contrast with the larger-sized organic materials in the fLF fraction (i.e., fLF-POM, Fig. 4ab), which show a high ^137^Cs retention capability, and may partly support the incorporation of ^137^Cs into the complex physical structures of larger-sized POM in the fLF fraction (mechanism (2), see above) as the main mechanism for ^137^Cs retention in the surface soil layers.

Our study demonstrates that, at least until 4 years after the accident, a significant amount of ^137^Cs has been retained in larger-sized POM (fLF-POM) in the surface layers of mineral soil in fLF-rich ecosystems. The results of the present study are consistent with the recent finding that a significant proportion (31–55%) of the ^137^Cs is present in association with macroaggregates (212–2000 μm) in forest surface soils affected by the Fukushima NPP accident^26^. Given that the ^137^Cs currently retained in the fLF fraction may be mobilized and become a source for ^137^Cs recycling in such ecosystems in the future, continuous and further investigations of the ^137^Cs distribution between the soil physical fractions, especially in fLF-rich ecosystems, are needed to improve our understanding of the ^137^Cs retention mechanism in the surface layers of the mineral soil. This is key to accurately assessing the long-term radioecological impacts of the Fukushima NPP accident.

## Methods

### Soil samples

Soil samples were collected from surface soil layers in July 2011 and July 2015 in the southwestern part of the city of Fukushima (37.71° N, 140.36° E), approximately 70 km northwest of the Fukushima Daiichi NPP^9,31^. The 2011 samples were from three sites under different land-use conditions (two cropland sites and a forest site). The cropland sites (CP-2 and CP-6) were a fallow field and an apple orchard, respectively, and the forest site (FR-4) was an evergreen coniferous forest dominated by Japanese cedar. The 2015 samples were from the FR-4 forest site. Note that the soil samples were collected after removing the vegetation and forest-floor litter layers. For more detailed information concerning the soil sampling procedure, site characteristics, and ^137^Cs contamination levels see Koarashi *et al*.^31^ and Matsunaga *et al*.^9^.

### Physical fractionation of the soil

Dried, sieved (<2 mm), and root-free (visible roots were removed by hand) soil samples were subjected to the following physical fractionation. The fractionation was based on the contrasting densities between soil mineral particles (typically 2.5–3.0 g cm^−3^) and organic materials (<1.4 g cm^−3^)^35,36^. Through fractionation, the soil samples were separated into a relatively mineral-free low-density fraction (fLF), a mineral-associated low-density fraction (mLF), and a high-density fraction (HF). This kind of method has been used for studying soil organic matter dynamics in the research fields of soil science and biogeochemistry^35–37,51–53^.

The soil samples (approximately 3–5 g) were weighed into 50-ml centrifuge tubes and mixed with 40 ml of a 1.6-g cm^−3^ sodium polytungstate (SPT) solution. The tubes were gently shaken and centrifuged at 2000 rpm for 30 min, and the floating materials (fLF) were aspirated onto a pre-baked glass microfiber filter (pore size: 0.7 μm). The cycle of gentle shaking, centrifugation, and aspiration was repeated until no fLF remained floating. The fLF fraction was then rinsed with ultrapure water on the filter and dried at 50 °C in an oven. The remaining soil samples were resuspended in 40 ml of 1.6-g cm^−3^ SPT in the centrifuge tubes, which were then sonicated in an ice bath for 5 min at 70% pulse for a total input of 300 J ml^−1^ (Sonifier 450, BRANSON, USA); the purpose of this process was to disrupt the aggregates and bonds between the low-density organic materials (mLF) and the high-density mineral particles (HF) in the SPT solution^35,36,53^. The mixture was centrifuged as before. The floating materials (mLF) were very fine and prone to clouding the supernatant, which often caused the filter to clog during aspiration^52,53^. To avoid this difficulty, mLF that was floating on the top of the SPT solution in the tubes was separately collected via pipetting prior to aspiration. The supernatant was then aspirated, and mLF in the supernatant was collected on a pre-baked glass microfiber filter. The cycle of centrifugation, pipetting, and aspiration was repeated until no mLF remained floating. The mLF collected via pipetting (mLF-p) was then rinsed with ultrapure water in a centrifuge tube with repeated centrifugation and dried at 50 °C in an oven. The mLF collected on the filter (mLF-f) was rinsed and dried as before. The mLF-p and mLF-f fractions were mixed to obtain whole mLF samples. After floating off the low-density fractions, the residue (HF) in the centrifuge tubes was rinsed more than five times with ultrapure water via shaking and centrifuging at 2000 rpm for 15 min and then dried at 50 °C in an oven.

The physical fractionation was conducted using three replicates for each of the soil samples, and the physical fractions (fLF, mLF, and HF) obtained from the three replicated samples were mixed together to obtain a sufficient amount of fractionated samples for the ^137^Cs analysis. The fractionated samples were photographed using a digital camera (WRYCOM-NF500, WRAYMER, Japan) mounted on a stereomicroscope (SZX7, OLYMPUS, Japan).

### Radiocesium analysis

A radiocesium analysis was conducted following Koarashi *et al*.^10^. The fractionated soil samples were ground into powder in a mortar, placed into plastic tubes, and analyzed for ^137^Cs using a well-type Ge detector (GWL-120, 230, ORTEC, USA). Measurement times were from 2,600 s to 420,000 s, depending on the ^137^Cs activity in the samples. The activity concentrations of the ^137^Cs were expressed in activity per unit dry weight (Bq kg^−1^ dw) and corrected for radioactive decay to the sampling date.

The ^137^Cs activity in each of the soil physical fractions (fLF, mLF, and HF) was estimated as

1 $$A=C\cdot M,$$

where *A* is the ^137^Cs activity (Bq) in the fraction, *C* is the ^137^Cs activity concentration (Bq kg^−1^) of the fraction, and *M* is the mass (kg) of the fraction obtained via physical fractionation. The difference in the soil mass between the unfractionated sample (i.e., prior to fractionation) and the sum of the three physical fractions (i.e., after fractionation) was considered to be the fraction lost during the fractionation procedure and represented <6% of the soil mass. The loss fraction was not analyzed for ^137^Cs in the present study, and therefore the amount of ^137^Cs in the loss fraction was not considered when evaluating the ^137^Cs distribution between the physical fractions of the soil.

### Further investigation into the fLF fractions

To explore the mechanisms for the high ^137^Cs retention in the fLF fractions, the fLF fractions were further separated into two fractions (fLF-POM and fLF-MP) via the following procedure.

A portion (approximately 0.2 g) of the fLF samples was put into a 50-ml beaker with 20 ml of ultrapure water and was then sonicated for 30 min in a water-bath sonicator (38 kHz, 80 W, tank volume: 1.6 L, US-1, SND Co. Ltd., Japan). The solution was transferred to a 50-ml centrifuge tube and mixed with 20 ml of a 2.8-g cm^−3^ SPT solution in the tube (i.e., the density of the solution was adjusted to 1.9 g cm^−3^). The tube was shaken well and then centrifuged at 2000 rpm for 30 min. Floating materials in the tube were collected as fLF-POM via pipetting. The remaining supernatant in the tube was discarded and the residue (fLF-MP: mineral particles with a density of >1.9 g cm^−3^) in the tube was rinsed with ultrapure water with repeated shaking and centrifugation. The fLF-POM collected via pipetting was also rinsed with ultrapure water in a tube with repeated centrifugation. Samples of fLF-POM and fLF-MP were dried at 50 °C in an oven, photographed, and analyzed for ^137^Cs in the same manner as mentioned above.

The fLF-POM and fLF-MP samples were ground into powder in a mortar, and their mineralogical compositions were measured using an X-ray diffractometer (Ultima IV, Rigaku, Tokyo) with CuKα radiation (40 kV, 20 mA). The XRD pattern was recorded over an angular range of 3–70° (2θ) with a step size of 0.03° and a counting time of 2 s per step. Minerals were identified using the Integrated X-ray powder diffraction software (PDXL Ver.2.4, Rigaku, Tokyo). The corresponding HF samples were also measured for comparison.

Finally, the fLF-POM and fLF-MP samples were analyzed for their total C and N content using an elemental analyzer (vario PYRO cube, Elementar)^39^.
